# Patients with Blunt Traumatic Spine Injuries with Neurological Deficits Presenting to an Urban Tertiary Care Centre in Mumbai: An Epidemiological Study

**DOI:** 10.5704/MOJ.1303.014

**Published:** 2013-03

**Authors:** Anoop C Dhamangaonkar, Deepak Joshi, Ravinish Kumar, Arvind B Goregaonkar

**Affiliations:** Department of Orthopaedics, Lokmanya Tilak Municipal General Hospital, Mumbai, India; Department of Orthopaedics, Lokmanya Tilak Municipal General Hospital, Mumbai, India; Department of Orthopaedics, Lokmanya Tilak Municipal General Hospital, Mumbai, India; Department of Orthopaedics, Lokmanya Tilak Municipal General Hospital, Mumbai, India

## Abstract

*Introduction:* Traumatic spine injuries are on the rise. The literature is sparse regarding epidemiology of patients with traumatic spine injuries from this part of the world. *Objectives:* To analyse the following in patients with traumatic spine injuries with neurological deficits: demographic and social profile, common modes of injury, pre-hospitalisation practices, region of spine affected, severity of neurological deficit and the lay individuals’ awareness about traumatic spine injuries. *Methods:* The study sample comprised 52 adult patients with traumatic spine injuries with neurological deficits. We collected data on demographic and social characteristics, mode of injury, pre-hospitalisation treatment, interval between injury and presentation, spine region affected and severity of neurological deficits and patient's knowledge about such injuries. *Results:* The average patient age was 31.32y. The male: female ratio was 2.25:1, and the most common modes of injury were fall from height, followed by traffic accident. More than half of the patients suffered cervical spine injuries, followed by dorsolumbar spine injuries. Only 9.61% of patients received pre-hospitalisation treatment. All patients understood there could be complete functional recovery after treatment for traumatic spine injuries. *Conclusion:* There is a growing need to improve railway and roadway safety equipment and to make it accessible and affordable to the susceptible economically weaker population. Attempts should be made to increase awareness regarding traumatic spine injuries.

**Key Words:**

Trauma; Spine; Deficit; Poor; safety; awareness

## Introduction

The incidence of traumatic spinal cord injuries has increased considerably over the past three decades^1^. These injuries occur as the result of road traffic accidents, fall of heavy objects, falls from height, falls into wells and construction sites2. The focus in the literature has often been on the orthopaedic management of such patients with little attention given to the epidemiology of patients presenting with neurological deficit to a tertiary care centre. In this case, epidemiological study includes attention to common modes of injury, demographic profiles of patients, social background of patients, region of spine affected, severity of injury and the lay individuals’ knowledge about traumatic spinal cord injuries. Exploration of these factors will reveal some important facts to help us identify the susceptible population groups and to take preventive measures. Increasing our knowledge of these existing gaps in the literature will help educate the lay population, health care personnel and industries (i.e., mass transit, railroads), alike about what measures should be taken to prevent, and primarily manage these patients with traumatic spine injuries.

## Aims and Objectives

1. To identify the demographic and social profile of patients with traumatic spine injuries with neurologic deficits.

2. To identify the common modes of traumatic spine injuries.

3. To identify common pre-hospitalisation on the field prehospitalisation practices for first aid traumatic spine injuries.

4. To identify the location of the spinal injury and the severity of the neurological deficits.

5. To assess the level of awareness of the lay public about traumatic spine injuries with neurologic deficits in terms of severity of injury, objectives of treatment and prognosis.

## Materials and Methods

After obtaining an institutional ethics committee approval and written informed consent, we recruited 52 adult patients with blunt traumatic spine injuries with neurological deficits, who were admitted to the orthopaedic trauma ward of this urban tertiary care centre over a 1½ year period in 2010-2011. This is a prospective, cross-sectional, observational, and epidemiological study. Patients with head injury, on psychiatric medications, with degenerative brain disease or younger than 18 years of age were excluded. Patients with polytrauma or with other associated injuries but with normal mentation at presentation were included in this study. We used a detailed questionnaire to collect data on demographic details such as age, sex, occupation, residence type and location, family structure, education, income, interval between time of injury and time of presentation to trauma centre, details of any treatment given in the field prior to hospitalization, region of spine affected, and common beliefs about traumatic spine injuries. We used Frankel’s grading to note the severity of neurological deficit at presentation (Table I). We did not pursue any follow-up in this sample population as the scope of this study is to focus on epidemiology and not outcomes.

## Results

The mean age of the patients was 32.32 years (range 19-60 year). The study included 36 males and 16 females male:female ratio 2.25:1. Thirty-two (61.53%) injuries resulted from a fall from height, 9 from railway train coach, 11 from an overhead loft, 8 from a ladder or staircase, and 4 from an unprotected roof. Eleven (21.15%) patients had sustained traumatic spine injuries following a road traffic accident (RTA) and 9 (17.30%) from a heavy object falling on the upper back. Among the 11 patients who were victims of road traffic accidents, 4 were two-wheeler drivers and all were wearing protective helmets at the time of injury. Seven patients were involved in a 4-wheeler accident, 4 of whom were travelling in a heavy motor vehicle without protective seat belts and the remaining 3 were travelling in a light motor vehicle in the front seats and wearing a seat belt. Fortyseven (90.39%) patients were natives of Mumbai. The chief catchment area of this tertiary care trauma centre was the urban township in and around Mumbai as well as the rural agricultural land of western Maharashtra. Twenty-eight patients (53.84%) were married. 22 (42.3%) were labourers. All lived with their nuclear family. Forty-four (65.38%) patients had not completed college. And 36 (69.23%) had an annual income less than Rs. 100000 (INR, Indian rupee). Forty-three (82.69%) patients presented within 6 hours of injury, but 47 (90.39 %) patients were not appropriately immobilised at the time of presentation. Among the 52 patients, 25 (48.07%) sustained cervical spine injuries (12 with Frankel’s grade A injury, 3 with grade B, 9 with grade C and 1 with grade D). Twenty-three (44.23%) patients were injured at dorsolumbar junction (5 patients with Frankel’s grade A injury, 8 with grade B, 7 with grade C and 3 with grade D). Four (7.69%) patients sustained an injury at the lower lumbar level (1 with Frankel’s grade A injury, 1 with grade B and 2 with grade C). Most patients were responsible for their own treatment expenses with only 21 patients receiving partial monetary aid for treatment from the institutional medical social service agencies. Fifteen (28.84%) were aware that this injury could lead to future disability, but all the patients thought that there would be complete functional recovery after appropriate treatment (Table II, summary)

## Discussion

Very little literature is available on the epidemiology of traumatic spine injuries in developing countries^3^. The average age of patients in our study, 31.32 years, was comparable to the existing literature in the Asian subcontinent and other developing countries^4^,^5^,^6^. Although recent papers report the predisposition of the elderly to traumatic spine injuries^7^, we did not have many elderly patients either because these cases did not present to our centre or due to an absence of neurological deficit in osteoporotic fractures following low energy falls. The male: female ratio in the present study was 2.25:1, lower than that of Chacko et al. in 1986 (male female ratio, 13.5:1)^8^ and Singh R et al. in 2001 (male: female ratio, 2.96:1)^2^, but comparable to recent reports from other developing countries^6^. The decreasing male to female ratio depicts the changing social trends with more women joining their male counterparts in outdoor activities.

Our findings of the percentage who fell from a height is comparable to a report by Leucht P et al.^9^. Among the patients who fall from height, a large proportion were falls from an overhead loft. The city of Mumbai is densely populated with growing number of slums. Shanties in these impoverished neighbourhoods are poorly built, many with insubstantial lofts built for want of space. People are forced to live in such hazardous circumstances as they cannot afford to buy well-constructed property. The other major cause of injury was due to a fall from a railway train coach, a mode not been reported earlier. Mumbai has a local railway system, which is one of the most commonly used modes of public transport. The trains are overcrowded during peak hours and hence these accidents occur. Improved public transport services may decrease the load on local railway services thereby decreasing the rate of such railway accidents.

Many reports have stated that RTAs are a very common mode of traumatic spine injuries^5^,^10^, but there has been a decrease in traumatic spine injuries from RTA due to improvements in safety equipment 11. In this study, RTA was responsible for 21.15% cases. Note that wearing seat belts and bicycle or motorcycle helmets without the use of air bags do not protect against cervical spine injuries. It is unfortunate to see that airbags and side impact bags are provided only in the high-end vehicles. Use of such basic road safety equipment should be made mandatory even in the basic vehicle models.

The 9 patients who sustained an injury due to fall of a heavy object, were loaders by profession who carried heavy loads on their shoulders and upper back. Any imbalance while carrying this heavy load can lead to flexion compression or a flexion distraction injury of the cervical spine. This points to the need for more mechanised loading and shifting facilities at various work sites.

We found no studies that mention the social background of patients affected with traumatic spine injuries. Most young patients fell at their work places or while commuting to or from their place of employment and most were poor, little literate Mumbai natives living with nuclear families. In this study, 43 (82.69%) patients presented to this tertiary centre within 6 hours of injury, an observation that is comparable to similar studies from other developing countries^12^, but lower than results seen from developed countries^13^. No Indian literature was found on similar lines. This lower timeframe to presenting at the hospital may be due to helpful bystanders who quickly move the victims to a hospital following an accident in a public place.

Considering these epidemiological and social factors in patients with traumatic spine injuries, there is a need to improve safety measures at workplaces, rail and road transport systems. Although transportation facilities have evolved and improved over the years, we found that lay individuals remain unaware of how to handle patients with traumatic spine injuries. The concept of on the field resuscitation, in-line immobilisation, use of spine boards, log-roll or lift and slide techniques and the potential risk of permanent neurological deficit due to improper handling are not discussed as part of public education through mass media. In our study, spine immobilisation was used in only 5 (9.61%) patients and only 3 (5.76%) patients were given primary treatment such as intravenous fluids and urinary catheterisation before they presented to us. Similar findings were reported in other studies from developing countries^4^,^12^.

Among patients with traumatic spine injuries, a majority had a cervical spine injury (48.07%) followed by dorsolumbar injuries (44.23%). We saw the majority of Frankel’s grade A injury in patients with cervical spine injury, similar to earlier reports^6^.

Ignorance about traumatic spine injuries is evident from the fact that only 15 (28.84%) patients were aware that this injury can lead to permanent disability and that all patients in this study thought that following definitive treatment, there would be complete functional recovery. This also underscores the need to improve the doctor-patient communication. Doctors themselves should be aware of this public unawareness about traumatic spine injuries. A diligent attempt must be made to increase the lay public’s awareness about traumatic spine injuries and the importance of primary treatment in such cases. These measures will go a long way to decrease the burden of traumatic spine injuries in society. Most patients had to bear treatment expenses themselves while some received partial aid with payment. Cost of these injuries includes expenses for diagnostics, surgical and rehabilitation treatment^14^. Considering this fact, there is now an emergence of government sponsored insurance for economically disadvantaged individuals in parts of India, including the locale of this study.

**Table I:**
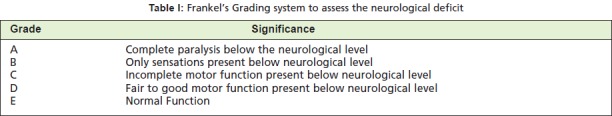
: Frankel’s Grading system to assess the neurological deficit

**Table II:**
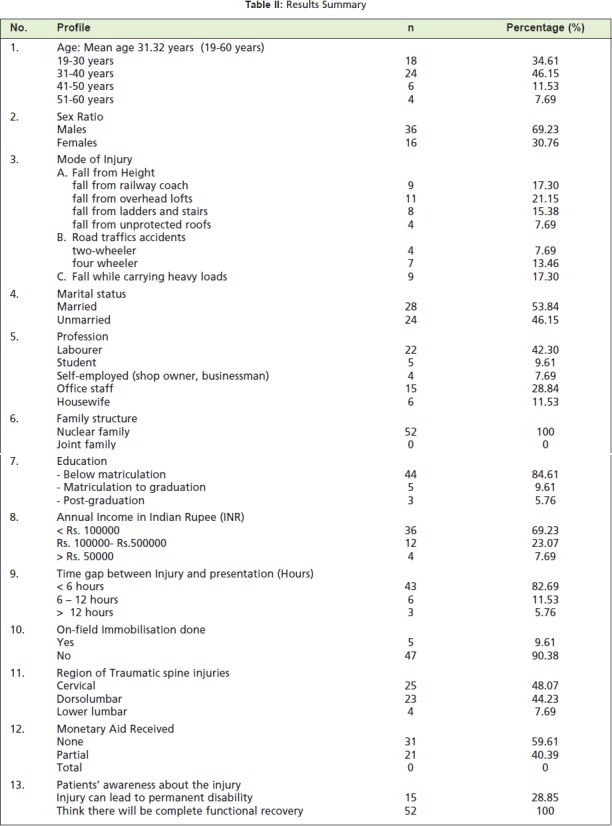
: Results Summary

## Conclusion

Traumatic spine injuries affect primarily the active, young, and productive population. Injury mechanisms include falls from height such as railway coaches, road traffic accidents and slipping while carrying heavy loads. Efforts are needed towards better and more affordable safety measures in housing facilities for the impoverished as well as railway and roadway systems. Use of seat belts and bicycle and motorcycle helmets do not protect against cervical spine injuries and resultant neurological deficits. There is also a need for improvements in transportation facilities and mechanised loading facilities at workplaces. An earnest attempt should be made to improve public awareness about traumatic spine injuries including severity of injury, appropriate handling techniques including basic primary treatment and prognosis after definitive final treatment. The number of paramedical personnel should be increased and they should be more available to administer primary treatment. A stronger social services network is needed to improve and speed rehabilitation of patients with traumatic spine injuries with neurological deficits.
